# Density of Healthcare Providers and Patient Outcomes: Evidence from a Nationally Representative Multi-Site HIV Treatment Program in Uganda

**DOI:** 10.1371/journal.pone.0016279

**Published:** 2011-01-17

**Authors:** Celestin Bakanda, Josephine Birungi, Robert Mwesigwa, Wendy Zhang, Amy Hagopian, Nathan Ford, Edward J. Mills

**Affiliations:** 1 The AIDS Support Organization, Headquarters, Kampala, Uganda; 2 British Columbia Centre for Excellence in HIV/AIDS, Vancouver, Canada; 3 Division of Infectious Diseases, University of Cape Town, Cape Town, South Africa; 4 Health Alliance International, School of Public Health, University of Washington, Seattle, Washington, United States of America; 5 Faculty of Health Sciences, University of Ottawa, Ottawa, Canada; University of Cape Town, South Africa

## Abstract

**Objective:**

We examined the association between density of healthcare providers and patient outcomes using a large nationally representative cohort of patients receiving combination antiretroviral therapy (cART) in Uganda.

**Design:**

We obtained data from The AIDS Support Organization (TASO) in Uganda. Patients 18 years of age and older who initiated cART at TASO between 2004 and 2008 contributed to this analysis. The number of healthcare providers per 100 patients, the number of patients lost to follow-up per 100 person years and number of deaths per 100 person years were calculated. Spearman correlation was used to identify associations between patient loss to follow-up and mortality with the healthcare provider-patient ratios.

**Results:**

We found no significant associations between the number of patients lost to follow-up and physicians (*p* = 0.45), nurses (*p* = 0.93), clinical officers (*p* = 0.80), field officers (*p* = 0.56), and healthcare providers overall (*p* = 0.83). Similarly, no significant associations were observed between mortality and physicians (*p* = 0.65), nurses (*p* = 0.49), clinical officers (*p* = 0.73), field officers (*p* = 0.78), and healthcare providers overall (*p* = 0.73).

**Conclusions:**

Patient outcomes, as measured by loss to follow-up and mortality, were not significantly associated with the number of doctors, nurses, clinical officers, field officers, or healthcare providers overall. This may suggest that that other factors, such as the presence of volunteer patient supporters or broader political or socioeconomic influences, may be more closely associated with outcomes of care among patients on cART in Uganda.

## Introduction

Most sub-Saharan African countries are struggling to improve the outcomes of HIV infected individuals by closing the gap between supply and demand of combination antiretroviral therapy (cART) and ensuring patient follow-up and adherence [1], [2]. A known key element of positive patient outcomes for cART is access to healthcare providers. In the majority of resource-limited sub-Saharan African countries, the limiting factor in cART scale-up and effective patient management is the deficit of qualified human resources for health [3], [4], [5]. The importance of having regular access to a healthcare provider is a frequently reported facilitator to cART adherence [6] and is a predictor of HIV viral suppression [7], [8], prevention of HIV resistance [9], HIV disease progression [10], and mortality [11].

Our present study uses data from a large nationally representative cohort of patients receiving cART in Uganda to examine the association between density of healthcare providers and patient outcomes. By testing this association, this study will provide insight for policy-makers on the health human resources considerations for cART service delivery. The results of this study are important to further understand the wide array of factors that influence patient outcomes of cART.

## Methods

### Ethics statement

This study received ethical approval from TASO Administrative Research Board, a Uganda National Science and Technology Council approved board, and from University of British Columbia. Informed consent was not required as this was routinely collected operational data and the institutional review boards waived the need for consent.

### Data Collection

Data were extracted from administrative records at The AIDS Support Organization (TASO). TASO is a nationwide non-governmental organization and provider of HIV/AIDS services in Uganda. Founded in 1987, it has supported over 200 000 HIV+ people and provides counselling, free access to cART, treatment for opportunistic infections, and active patient retention strategies to reduce the number of patients lost to follow-up. TASO operates in services sites distributed across Uganda. Ten sites were included in this study: Entebbe, Gulu, Jinja, Masaka, Mbale, Mbarara, Masindi, Mulago, Soroti, and Tororo.

All 23 747 patients 18 years of age and older who initiated cART at the ten selected TASO sites between January 1, 2004 and December 31, 2009 contributed to this study. Patients were followed until death, loss to follow-up, or end of study period, whichever occurred first. Healthcare providers use standardized forms at each clinic visit to detail patients' demographic, clinical, psychosocial, and drug use data. These data are then entered into the TASO data collection database at each site by trained data capturers. All patient data are anonymized through the use of unique, confidential identification numbers. Healthcare provider density data were obtained from human resource records at TASO service sites.

### Data Analysis

The following healthcare providers were included in this study: physicians, nurses, clinical officers, and field officers. Clinical officers function as physician's assistants and work in the service sites, while field officers travel to surrounding communities tracking patients using active retention strategies, distributing cART, and providing additional patient support.

For each service site, we calculated physician-patient ratio, nurse-patient ratio, clinical officer-patient ratio, field officer-patient ratio, and healthcare provider-patient ratio (which included all four types of healthcare providers). All ratios were calculated by summing the number of providers, dividing it by the summed number of patients over the four-year study period, and multiplying by 100 to give the number of healthcare providers per 100 patients. We considered patients that were unaccounted as having been lost to follow up, thus we did not have an issue with missing data.

We used two rates as indicators of patient outcome: number of patients lost to follow-up per 100 person years and number of deaths per 100 person years over the study period. Number of patients lost to follow-up per 100 person years was calculated by dividing the total number of patients lost to follow-up by the total number of patient years over the study period for each service site and then multiplying by 100. The number of deaths per 100 person years was calculated by dividing the total number of patient deaths by the total number of patient years over the study period for each service site and then multiplying by 100.

Spearman correlation coefficients were calculated to identify associations between the outcome variables (patient loss to follow-up and mortality) with healthcare provider-patient ratios by TASO service sites. The Spearman correlation coefficient was used because it is a non-parametric test that does not require a normal distribution. All tests of significance are two sided and *p*<0.05 indicates a statistically significant association. All analyses were conducted using SAS version 8 (SAS Institute, Cary, NC).

## Results

Table 1 shows the ratio of healthcare providers to patients by service site. The healthcare provider-patient ratios varied across the ten TASO service sites. The greatest range was in physician-patient ratios, from 0.22 in Masindi to 1.77 in Masaka. Overall, sites had a mean of 0.94 physicians per 100 patients. Nurse-patient ratios ranged from 1.20 in Entebbe to 2.16 in Masaka with a mean of 1.59 nurses per 100 patients. Clinical officer-patient ratios ranged from 0.10 in Masaka to 0.28 in Tororo with a mean of 0.16 clinical officers per 100 patients. Field officer-patient ratios ranged from 0.23 in Mulago to 0.65 in Gulu with a mean of 0.38 field officers per 100 patients. Overall, the summed ratio of all healthcare providers to patients varied from 2.48 in Entebbe to 4.83 in Masaka. The mean number of healthcare providers per 100 patients was 3.64.

**Table 1:** Ratio of healthcare providers to patients measured as the number of providers per 100 patients according to service site.

Site	Physician-patient ratio	Nurse-patient ratio	Clinical officer-patient ratio	Field officer-patient ratio	All healthcare providers-patient ratio
**Entebbe**	0.70	1.20	0.12	0.45	2.48
**Gulu**	1.02	1.75	0.23	0.65	4.57
**Jinja**	1.00	1.66	0.05	0.33	3.26
**Masaka**	1.77	2.16	0.10	0.39	4.83
**Mbale**	0.61	1.33	0.14	0.25	2.88
**Mbarara**	1.07	1.69	0.11	0.24	3.54
**Masindi**	0.22	1.51	0.22	0.29	3.09
**Mulago**	1.36	1.55	0.15	0.23	3.89
**Soroti**	0.99	1.34	0.16	0.53	3.68
**Tororo**	0.69	1.70	0.28	0.41	4.19
**Overall**	0.94	1.59	0.16	0.38	3.64

Table 2 shows the number of patients lost to follow and the number of deaths by service site. The number of patients lost to follow-up per 100 person years varied by service site with a mean of 1.53 patients lost to follow-up per 100 person years across all sites. Soroti had the lowest number of patients lost to follow-up at 0.53 patients per 100 person years. In contrast, Entebbe had the highest with 2.24 patients lost to follow-up per 100 person years. The number of deaths per 100 person years also varied by service site: Entebbe had the lowest number of deaths per 100 person years at 0.64, while Mbale had the highest at 2.42 deaths per 100 person years. The mean number of deaths per 100 person years was 1.36 patients.

**Table 2:** Number of patients lost to follow up per 100 person years and crude mortality rate according to service site.

Site	Number of patients lost to follow-up per 100 person years	Number of deaths per 100 person years
**Entebbe**	2.24	0.64
**Gulu**	2.08	1.03
**Jinja**	1.55	1.76
**Masaka**	0.82	1.92
**Mbale**	0.75	2.42
**Mbarara**	2.11	0.96
**Masindi**	1.20	1.69
**Mulago**	2.19	1.03
**Soroti**	0.53	1.02
**Tororo**	1.80	1.17
**Overall**	1.53	1.36

**Figure 1:**
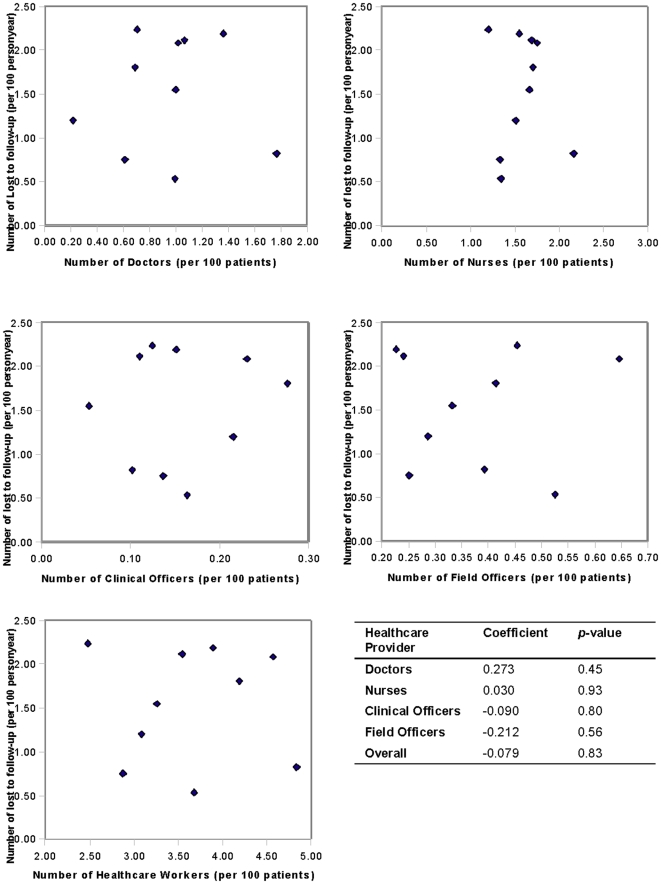
Associations between the number of patients lost to follow-up per 100 person years and the number of healthcare providers per 100 patients. X axis represents number of health workers (per 100 patients), y axis represents number of events (per 100 patients).

**Figure 2:**
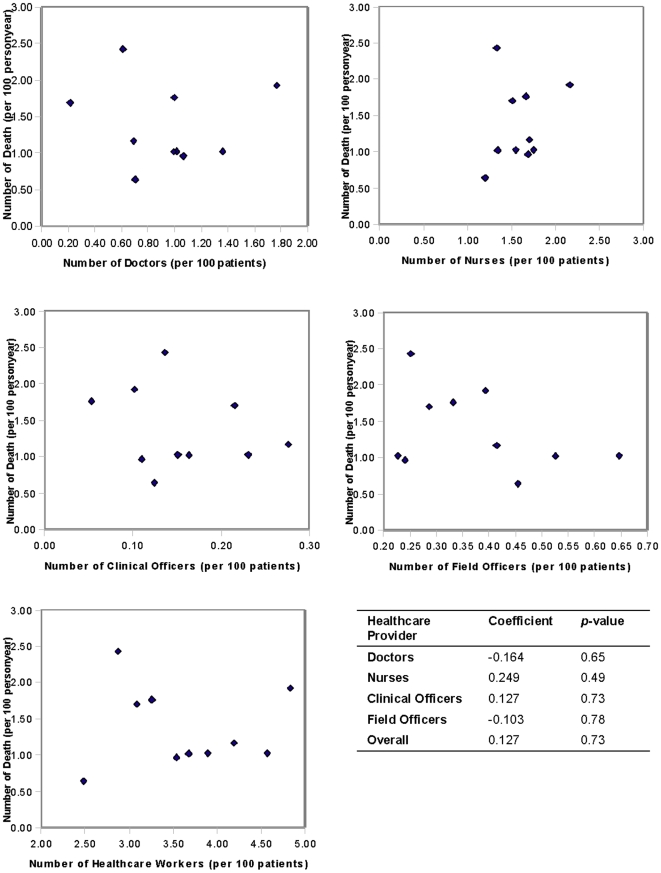
Associations between the number of deaths per 100 person years and the number of healthcare providers per 100 patients. X axis represents number of health workers (per 100 patients), y axis represents number of events (per 100 patients).

Figure 1 shows the associations between the number of patients lost to follow-up per 100 person years and the number of healthcare providers per 100 patients. No significant associations were observed between the number of patients lost to follow-up and physicians (*p* = 0.45), nurses (*p* = 0.93), clinical officers (*p* = 0.80), field officers (*p* = 0.56), and healthcare providers overall (*p* = 0.83). Figure 2 shows the associations between mortality per 100 person years and the number of healthcare providers per 100 patients. No significant associations were observed between mortality and physicians (*p* = 0.65), nurses (*p* = 0.49), clinical officers (*p* = 0.73), field officers (*p* = 0.78), and healthcare providers overall (*p* = 0.73).

## Discussion

Our study examined the associations between healthcare provider density and outcomes of care using data from a large nationally representative cohort of patients receiving cART in Uganda. Patient outcomes, as measured by loss to follow-up and mortality, were not significantly associated with the number of doctors, nurses, clinical officers, field officers, or healthcare providers overall.

Although healthcare provider density has been shown to influence patient outcomes and health indicators in cross-country examinations [12], [13], this was not the case among patients attending TASO clinics in Uganda. It is likely, however, that the relationship between healthcare provider density and patient outcomes is linked closely with country- or community-level characteristics and cohort attributes. We cannot know the extent of generalizability of our study as cART programs in other countries infrequently provide outcomes on health worker status and clinical outcomes. A recent study by Lambdin et al, in Central Mozambique, found that patients attending clinics with larger patient volumes and a larger number of patients per pharmacy staff had a higher risk of patient attrition (defined as lost to follow-up or death) [14]. This association, however, was not significant when the authors considered the number of patients per clinicians [14].

At TASO, it is probable that the density of volunteers available to patients is associated with outcomes of care. TASO uses a community-based model of support for patients receiving cART, where each patient has a supporter, often a family member or friend, who provides interpersonal support and reminds the patient to adhere to their cART regime. Given that the presence of such social supports is a widely accepted facilitator of positive patient outcomes [6], the presence of such a supporter may be a better predictor of patient outcome than the presence of formal healthcare providers in this setting. However, the TASO database does not include data on the density of community-based supporters, so its relationship to patient outcomes could not be examined.

The link between healthcare provider density and patient outcomes in the TASO setting are likely influenced by additional factors not captured in the database. For example, broad socioeconomic and political factors vary between sites. For example, during the study period, Northern Uganda was facing a civil war, particularly affecting the Gulu region [15]. While evidence shows that HIV treatment can be effectively provided in such settings [16], [17], variations in political and socioeconomic factors may influence healthcare provider retention and patient loss to follow-up and mortality. The interplay between these such factors with HIV service delivery is important and well accepted [18], but cannot be analysed given the data available.

Despite the lack of significant relationship between patient outcomes and healthcare provider density, the study also revealed that across service sites there exists variation among these variables. Across all ten sites, healthcare providers are in short supply and half of the sites report that unmet demand for healthcare workers is a key challenge to provision of care.

As with any study of this nature there are several limitations to consider. First, the TASO database does not include information on HIV viral load or HIV resistance, which would have been important indicators of HIV disease progression to consider. Also, it was not possible to include CD4 cell count as a patient outcome in this study because the TASO database does not include complete longitudinal CD4 cell count data. This lack of complete CD4 cell counts is a reflection of the diverse settings in which TASO works in Uganda. This problem is common in other resource-constrained settings as well [19]. Our study did not demonstrate a relationship between the density of healthcare workers and either loss to follow up or mortality. It is possible that our study was hampered by the number of centres, rather than the number of patients, that may have restricted variance. It is possible that with more centres we would have observed a different finding.

We created indicators of healthcare providers and outcomes. Although density of health workers is a well-established indicator, there are both pros and cons to applying this as a rate. We considered all health cadres individually and also a pooled group. We have no doubt that some providers may have greater skills than others. Similarly, providing AIDS care may have been more complicated during the early days of cart provision (eg. 2004) when those initiated onto treatment were typically very sick. This may have changed over time. Also, our mortality rate addresses known deaths. Although TASO has a low rate of loss to follow up, we would expect that some patients that were lost had, in fact died, thus our mortality rates may underplay mortality rates. Because our study examined both attrition and mortality separately, and because TASO has active retention of patients, we believe our findings are broadly applicable. In studies examining mortality outcomes as a primary outcome, we typically assume that 50% of those lost to follow up have died.

The quality of the data and outcomes of this study are strengthened by the large sample size and the multiple years of data available for analysis. The cohort also represents cART patients nationwide, thus capturing a wide range of differing patient experiences and allowing for regional comparisons. TASO offers services across all regions of Uganda, though specific TASO centres or through Ministry of Health/TASO supported centres in more rural settings. While other providers are available in Uganda, the cART regimens offered are similar and the laboratory services offered are similar. Further, the use of community-based patient supporters to facilitate adherence, as the literature suggests [20], [21], contributed to high adherence rates in TASO patients [22], a key determinant of patient outcomes. The TASO cART delivery model also incorporates active retention strategies to locate patients who do not attend their scheduled appointments, thus reducing the degree of loss to follow-up [23]. In this cohort, 93% of patients were retained over the four years. In contrast, a systematic review of 32 ART programs in Africa showed that, on average, patient retention rates are 61.6% over a two year period [23].

Effectively providing HIV/AIDS treatment in resource-limited settings requires careful consideration of the health human resources, including the availability of healthcare providers. Policy or spending decisions in this area can have a direct impact on patient clinical outcomes. The results of the present study indicated that there were no significant associations between patient outcomes and healthcare provider density in Uganda. This suggests that that other factors, such as the presence of volunteer patient supports or broader political or socioeconomic influences, may be more closely associated with outcomes of care among patients on cART in this setting.
